# LLM-optimized wavelet packet transform for synchronous condenser fault prediction

**DOI:** 10.1371/journal.pone.0330429

**Published:** 2025-08-29

**Authors:** Dongqing Zhang, Chaofeng Zhang, Michel Kadoch, Tao Hong, Shenglong Li, Wenqiang Zhao

**Affiliations:** 1 DC Technical Center of State Grid Corporation of China, Beijing, China; 2 State Grid Hunan Extra High Voltage Substation Company and Substation Intelligent Operation and Inspection Laboratory of State Grid Hunan Electric Power Co., Ltd., Changsha, China; 3 Cross-Strait Tsinghua Research Institute (CTRI), Xiamen, Fujian, China; 4 School of Electronics and Information Engineering, Beihang University, Beijing, China; 5 State Grid Qinghai Electric Power Research Institute, Xining, China; University of Botswana Faculty of Engineering and Technology, BOTSWANA

## Abstract

This paper proposes an innovative approach for predicting faults in synchronous condensers in ultra-high voltage direct current (UHVDC) transmission systems. The framework combines Wavelet Packet Transform (WPT) for intelligent feature extraction with an enhanced Gated Recurrent Unit (GRU) network augmented by multi-head attention mechanisms. WPT is employed for efficient decomposition of fault signals into multiple frequency sub-bands, facilitating the extraction of fault features such as energy, entropy, and statistical moments. By applying Large Language Models (LLM) to WPT, an intelligent feature selection mechanism significantly improves both detection accuracy and processing efficiency. The Multi-Head Attention GRU (MHA-GRU) network architecture is designed to capture complex temporal dependencies in fault signals while maintaining computational efficiency. Comprehensive experimental results demonstrate that our framework consistently outperforms state-of-the-art methods across all performance metrics, including classification accuracy, detection time, and false alarm rate. The system exhibits robust stability under varying load conditions with particularly significant improvements in air-gap eccentricity fault detection. The proposed approach provides a reliable solution for early fault prediction in UHVDC synchronous condensers, enabling timely maintenance intervention before minor issues develop into critical failures.

## 1 Introduction

In ultra-high voltage direct current (UHVDC) transmission networks, synchronous condensers serve as vital equipment for maintaining system stability through dynamic reactive power management. Synchronous condensers are essential components in UHVDC transmission systems, where they provide critical dynamic reactive power support. This support plays a significant role in stabilizing voltage levels across the network, thereby ensuring overall grid stability. These machines are fundamental in regulating voltage transmit substantial power over extended distances is a key feature of UHVDC systems, and maintaining consistent voltage levels through precise reactive power control is essential for optimal operation [1].

Long-distance power delivery through UHVDC systems presents unique operational challenges. The need for synchronous condensers in these systems becomes even more pronounced given the challenges associated with long-distance power transmission. UHVDC systems often face fluctuations in power demand and varying load conditions, which can cause voltage instability. Synchronous condensers respond rapidly to these changes by absorbing or supplying reactive power as needed, thereby counteracting potential disturbances and maintaining voltage balance. The network must cope with dynamic load variations and shifting power requirements, which can lead to voltage fluctuations. To address these issues, synchronous condensers act as rapid-response units, automatically adjusting their reactive power output to neutralize voltage deviations and preserve system equilibrium. This swift compensation mechanism helps prevent voltage instability and ensures reliable power transfer throughout the network. The stability of UHVDC transmission systems heavily depends on sufficient reactive power compensation. Without proper support, these systems become vulnerable to voltage instability that can cascade into complete voltage collapse scenarios[1].

Fault prediction in synchronous condensers is a critical aspect of maintaining operational safety and preventing catastrophic failures across the power grid. These devices play a key role in stabilizing voltage levels in UHVDC transmission systems, and any malfunction can have serious consequences, potentially leading to widespread outages. Therefore, the ability to detect and predict faults at an early stage is essential to ensure the smooth and reliable operation of the entire transmission network. These machines perform the critical function of voltage stabilization in UHVDC networks [2]. Their optimal performance directly impacts the entire transmission system’s health. Prompt identification of developing issues, particularly rotor winding short circuits or air-gap eccentricity, enables maintenance teams to implement timely interventions before minor problems escalate into severe system failures. For instance, a rotor winding short circuit can reduce the effective number of winding turns, compromising the machine’s ability to generate the necessary magnetic field for reactive power support. Similarly, air-gap eccentricity, which occurs when the rotor deviates from its central axis, can cause uneven magnetic fields and excessive vibrations, leading to mechanical stress and potential long-term damage. When synchronous condensers malfunction, consequences range from localized power quality issues to system-wide blackouts with significant economic impact. Proactive monitoring is therefore essential, as effective fault prediction systems allow operators to address developing issues before they become critical failures, protecting network integrity and ensuring reliable power delivery.

Existing approaches for fault diagnosis and prediction in electrical rotating machinery can be broadly categorized into traditional methods, signal processing techniques, and artificial intelligence-based solutions. Traditional methods often rely on visual inspections, thermal imaging, and vibration analysis, which are largely reactive in nature and limited by their inability to detect incipient faults.

Signal processing techniques, particularly those based on wavelet transforms, have shown significant potential in fault diagnosis applications due to their ability to analyze non-stationary signals at multiple scales. Modern research demonstrates that comprehensive diagnostic approaches combining Wavelet Packet Transform (WPT) with intelligent algorithms and continuous monitoring capabilities are crucial for effective fault detection and classification in complex systems. WPT is particularly effective in decomposing fault signals into multiple frequency sub-bands, making it easier to extract key fault features like energy, entropy, and statistical moments that may indicate faults. These tools can analyze complex patterns in operational data, detect anomalies, and provide early warnings, enabling operators to address potential issues before they escalate into major system failures. However, these approaches often require manual parameter selection, introducing subjective judgments that impact feature extraction quality.

Wavelet packet decomposition (WPD) has proven valuable for extracting distinctive features from complex signals in fault diagnosis applications. Recent research showcases its effectiveness: [3] developed a WPD-based weighted multi-scale fractional permutation entropy approach for railway equipment fault diagnosis with improved accuracy; [4] integrated variational mode decomposition with multi-scale analysis and the ReliefF algorithm for enhanced vibration-based fault detection; and [5] created a derivative multi-scale entropy method that captures complementary fault information and employs decision fusion strategies to improve classification performance.

Artificial intelligence has transformed fault diagnosis through advanced data processing capabilities. Deep learning architectures excel at identifying subtle system anomalies, with Radial Basis Function neural networks [6] particularly effective at fault classification by modeling complex non-linear relationships in operational data.Gated Recurrent Unit (GRU) networks are a type of recurrent neural network (RNN) designed to handle sequential data, making them well-suited for analyzing time-series data generated by synchronous condensers. These networks excel in capturing temporal dependencies and managing non-linear dynamics, which are often present in the operational signals of electromechanical systems. Unlike traditional RNNs, GRUs are more efficient due to their simplified architecture, which reduces computational complexity while maintaining the ability to model long-term dependencies [7]. Despite these advantages, computational complexity remains a challenge, with many AI systems requiring substantial resources that can create bottlenecks in real-time monitoring applications.

Despite these advancements, existing fault diagnosis approaches for synchronous condensers face several challenges. First, most conventional signal processing methods require manual parameter selection for wavelet decomposition, introducing subjective judgments that can significantly impact feature extraction quality. Second, the features extracted through traditional methods often contain redundant information, increasing computational complexity without proportional gains in diagnostic accuracy. Third, existing neural network models struggle to simultaneously capture both short-term anomalies and long-term degradation patterns in synchronous condenser operational data [8].

The complexity of synchronous condensers, which are sophisticated electromechanical devices, further underscores the need for advanced diagnostic tools. Unlike traditional machinery, synchronous condensers operate under varying conditions, handling fluctuating loads and dynamic power requirements. This variability makes it difficult to identify faults using basic diagnostic methods, as the symptoms may not always be immediately apparent. The intermittent nature of fault symptoms necessitates the implementation of advanced diagnostic technologies that can continuously monitor system parameters and detect subtle changes indicative of developing faults [9]. Modern approaches incorporating Wavelet Packet Transform (WPT), artificial intelligence, and continuous monitoring systems have proven crucial for reliable fault prediction and diagnosis. WPT is particularly effective in decomposing fault signals into multiple frequency sub-bands, making it easier to extract key fault features like energy, entropy, and statistical moments that may indicate faults. These tools can analyze complex patterns in operational data, detect anomalies, and provide early warnings, enabling operators to address potential issues before they escalate into major system failures.

The primary objective of this study is to design and develop an innovative and comprehensive framework for fault prediction in synchronous condensers, specifically those operating within UHVDC transmission systems. Given the critical role of synchronous condensers in maintaining grid stability by providing dynamic reactive power support, ensuring their reliable operation is essential. However, these machines are prone to a range of faults that, if left undetected, can lead to significant disruptions in power transmission. This study aims to address these challenges by proposing a robust fault prediction system that can detect and diagnose potential issues at an early stage, thereby enhancing the reliability and safety of UHVDC networks. To address these challenges, this research introduces an innovative predictive maintenance system with several key contributions. First, we propose a novel framework that integrates LLMs with wavelet packet transform for intelligent feature selection, eliminating the need for manual parameter tuning and significantly improving feature discrimination. Second, we develop an enhanced GRU network augmented by multi-head attention mechanisms to effectively capture complex temporal dependencies in fault signals. Third, we demonstrate superior performance in fault classification accuracy, detection time, and false alarm rate through comprehensive experimental validation.

The proposed solution’s strength lies in its ability to handle extensive datasets while maintaining real-time monitoring capabilities. When anomalies are detected, the system promptly notifies maintenance personnel, allowing for proactive interventions before minor issues develop into major failures. This advancement represents a crucial step forward in UHVDC system maintenance, offering power utilities a reliable tool to maximize operational efficiency and minimize downtime through early fault detection and prevention.

The remainder of this paper is organized as follows: Sect 2 reviews related work and existing methods in synchronous condenser fault diagnosis. Sect 3 presents the theoretical foundation of wavelet packet transform for fault prediction. Sects 4 and 5 detail our proposed framework, including the LLM-enhanced feature extraction and neural network model design. Sect 6 presents experimental results, and Sect 7 concludes the paper.

## 2 Related work

### 2.1 Synchronous condenser fault prediction: Current status and challenges

Synchronous condensers provide critical reactive power compensation in UHVDC systems, operating under unique conditions with frequent load variations and complex electromagnetic interactions.

Research on synchronous condenser fault prediction has developed as a specialized field. Study [10] examined loss of excitation impacts on UHVDC systems, establishing specific fault indicators. Unlike conventional rotating machinery, synchronous condensers require specialized approaches as highlighted by [11], which demonstrated how these devices must be designed for transient fault currents in renewable energy grids.

Recent advances integrate wavelet transforms with machine learning. Research [12] developed a discrete wavelet transform method for power system fault detection applicable to synchronous condenser diagnostics, offering advantages over traditional Fourier-based approaches.

Our work addresses these limitations by introducing a framework specifically designed for the unique characteristics of synchronous condenser fault patterns, leveraging large language models to optimize wavelet packet transform parameters for enhanced feature extraction.

### 2.2 Existing fault diagnosis and prediction methods wavelet packet transform

While established monitoring practices for synchronous condensers have their place, they come with significant drawbacks. Conventional methods such as visual examinations and thermal assessments primarily function as reactive tools, identifying problems only after damage becomes apparent. This delayed detection often results in extended equipment downtime and significant maintenance expenses.

The deployment of infrared technology for heat pattern analysis can reveal problems like deteriorating insulation and excessive temperature buildup. Similarly, physical inspections help identify visible issues such as component degradation, oxidation, and improper alignment. Yet these approaches have inherent limitations - their effectiveness heavily depends on inspector expertise, and subtle deterioration in early stages often goes unnoticed. Furthermore, intermittent issues that don’t present constant symptoms pose a particular challenge, as they may not manifest during scheduled maintenance checks.

A key shortcoming of these traditional techniques is their inability to provide continuous monitoring. By capturing only isolated moments in time, they fail to track progressive changes in equipment condition. This makes it challenging to anticipate and prevent potential failures before they become critical. Moreover, these inspection methods typically require equipment shutdown and considerable manual effort, making them impractical for ongoing system monitoring. This gap in real-time oversight capabilities represents a significant limitation in conventional diagnostic approaches.

Recent advances in diagnostic technology have transformed the maintenance landscape through the integration of sophisticated sensor networks and artificial intelligence. These contemporary systems enable continuous parameter tracking, including thermal conditions, mechanical vibrations, and electrical flow patterns. By leveraging real-time data analysis, maintenance teams can identify emerging issues and implement preventive measures before critical failures occur.

The field of signal processing has introduced particularly powerful diagnostic tools for synchronous condenser monitoring. Among these, WPA and Weighted Kurtogram Analysis represent leading analytical techniques, offering enhanced capabilities for detecting subtle mechanical and electrical anomalies. These methodologies significantly surpass traditional monitoring approaches by conducting comprehensive signal examination, particularly useful for identifying bearing wear and rotor position irregularities.

The distinctive strength of WPA lies in its capacity to handle dynamic, non-stationary signal patterns characteristic of electromechanical equipment. Through its multi-band frequency decomposition approach, WPA excels where conventional Fourier methods fall short, enabling the detection of nascent mechanical issues such as subtle vibrational anomalies. Complementing this, Weighted Kurtogram Analysis employs statistical kurtosis measurements to identify signal irregularities, specifically targeting frequency ranges where sharp peaks may indicate developing mechanical defects, such as bearing damage or rotor misalignment.

Despite their sophistication, signal processing diagnostic approaches face several challenges. A key constraint is the requirement for manual data feature selection and extraction, which introduces potential human error and scalability issues, particularly in real-time applications. Moreover, these systems are vulnerable to various interference sources, including electrical noise, mechanical disturbances, and ambient conditions. While signal filtering and preprocessing techniques can help mitigate these issues, complete elimination of diagnostic inaccuracies remains challenging.

The emergence of artificial intelligence has revolutionized fault detection capabilities in synchronous condensers and similar electromechanical systems. Machine learning algorithms, particularly deep learning architectures, demonstrate remarkable ability to process extensive datasets and identify subtle system irregularities. These AI-driven solutions maintain high accuracy even in environments with significant background noise and operational complexity.

Among the notable AI implementations, Radial Basis Function (RBF) neural networks have proven particularly effective at fault classification. Their strength lies in managing complex, non-linear relationships within operational data, enabling accurate identification of various system malfunctions, from rotor positioning issues to stator abnormalities [13]. In parallel, Gated Recurrent Unit (GRU) networks bring specialized capabilities for sequential data analysis. Their streamlined architecture facilitates efficient processing of temporal patterns, enabling both historical trend analysis and real-time fault prediction while maintaining computational efficiency.

Processing limitations constitute another significant barrier to implementation. The complex nature of these AI systems demands considerable computational resources, which can create bottlenecks in real-time monitoring scenarios. Although recent developments in computing infrastructure have addressed certain technical limitations, the field continues to seek improvements in two critical areas: algorithmic optimization and streamlined data management protocols.

### 2.3 Existing fault diagnosis and prediction methods wavelet packet transform

As discussed in previous sections, various approaches have been developed for synchronous condenser fault diagnosis and prediction. Table 1 provides a comprehensive comparison of these methods, highlighting their advantages, limitations, and application domains.

**Table 1:** Comparison of existing fault diagnosis and prediction methods for synchronous condensers.

Reference	Method	Advantages	Limitations	Application
Ma et al. [1]	Multi-level federated learning	Distributed processing, Privacy preservation	High computational complexity, Communication overhead	General fault diagnosis
Wang et al. [2]	Synchronous condenser for SSO mitigation	Effective for sub-synchronous oscillations	Limited to specific fault types	PMSG-based wind farms
Hsu et al. [3]	Solid-state excitation	Improved response time, Enhanced stability	Complex implementation, Higher cost	Power system stabilization
Li et al. [14]	Feature extraction-based analysis	Comprehensive fault coverage	Manual feature engineering required	Semiconductor manufacturing
Ren et al. [15]	Wavelet Packet Transform with domain adaptation	Cross-domain applicability	Limited real-time capability	Rolling-bearing diagnosis
Hou and Han [13]	RBF neural network	Good generalization ability	Requires extensive training data	Function approximation
Traditional methods	Visual inspection, Thermal imaging	Simple implementation	Reactive approach, Intermittent fault limitation	Basic maintenance
Conventional signal processing	Fourier analysis, Time-domain analysis	Established methodologies	Poor non-stationary signal handling	Stable operational conditions
Our approach	LLM-enhanced WPT with MHA-GRU	Superior fault classification accuracy, Fast response time, Computational efficiency	Requires initial training with historical data	UHVDC synchronous condenser fault prediction

As shown in Table 1, existing methods for synchronous condenser fault diagnosis and prediction exhibit various limitations that our proposed framework effectively addresses. Traditional approaches rely heavily on reactive monitoring and lack real-time capabilities, while conventional signal processing techniques struggle with non-stationary signals characteristic of developing faults. Recent AI-based methods have improved detection capabilities but often require extensive computational resources or manual feature engineering. Our proposed framework integrates LLM-enhanced Wavelet Packet Transform for intelligent feature selection with a Multi-Head Attention GRU network for efficient temporal pattern analysis, providing automatic optimization of wavelet packet decomposition parameters that eliminates subjective judgments in manual configuration. This novel approach enhances feature extraction to significantly improve discrimination between different fault types while maintaining computational efficiency through the streamlined MHA-GRU architecture, enabling real-time processing even under high system loads. The results demonstrate superior fault classification accuracy across various fault types including rotor, air-gap, and stator faults, with rapid fault detection and minimal delay, allowing for timely maintenance intervention in UHVDC transmission systems.

## 3 Theoretical foundation and synchronous condenser operation

This section establishes the theoretical framework for synchronous condenser fault detection. We present the operating principles of these devices within UHVDC systems, followed by the wavelet packet transformation methodology that underpins our approach. The discussion examines fault characteristics and their physical manifestations, detailing major fault types and their mechanisms. This theoretical foundation sets the stage for our proposed LLM-enhanced fault prediction system.

### 3.1 Wavelet packet transform

Unlike traditional Wavelet Transform methods, the Wavelet Packet Transform provides an enhanced approach to signal analysis through comprehensive frequency decomposition. The key distinction lies in its processing methodology - WPT conducts recursive splitting of both high and low-frequency bands, creating a more granular representation of the signal across multiple frequency levels. In contrast, conventional WT only breaks down the low-frequency components, limiting its analytical depth.

This comprehensive decomposition technique enables WPT to extract and analyze subtle signal characteristics that might otherwise remain undetected. Such capability makes it particularly valuable in specialized applications where detailed signal analysis is crucial, such as identifying system malfunctions, eliminating unwanted noise from signals, and conducting sophisticated time-frequency examinations. Let *x*(*t*) represent the continuous-time signal to be analyzed. The initial step in the WPT involves decomposing this signal using a pair of filters: a low-pass filter *h*(*t*) and a high-pass filter *g*(*t*). In matrix form, the convolution operation can be expressed as:

$$a=\beta W_{g}x$$ (1)

$$b=\beta W_{h}x$$ (2)

where *β* is the scaling factor (typically $\beta =\frac{1}{\sqrt{2}}$ for energy preservation), and *W*_*g*_, *W*_*h*_ are the convolution matrices constructed from the filter coefficients.

$$W_{g}=[\begin{array}{ccccc} g(1) & g(0) & 0 & 0 & \cdots \\ g(2) & g(1) & g(0) & 0 & \cdots \\ g(3) & g(2) & g(1) & g(0) & \cdots \\ \vdots & \vdots & \vdots & \vdots & \ddots \end{array}]$$ (3)

The signal reconstruction can then be represented as:

$$x=\beta \left(W_{g}^{T}\cdot a+W_{h}^{T}\cdot b\right)$$ (4)

where $W_{g}^{T}$ and $W_{h}^{T}$ are the transpose of the filter matrices, ensuring perfect reconstruction when the filters satisfy the quadrature mirror filter conditions.

The iterative decomposition extends through *L* levels, creating a hierarchical structure that captures all frequency sub-bands. Below illustrates the two-level decomposition tree structure.

x(t)→{LL,LH,HL,HH}→{LLL,LLH,LHL,LHH,…} (5)

Unlike conventional wavelet transforms which split signals into just two bands, WPT offers superior analytical capabilities through comprehensive frequency band decomposition. Traditional WT produces basic high and low-frequency separations, but this binary approach often proves inadequate when examining complex signals, particularly those associated with system malfunctions. The distinguishing strength of WPT emerges from its iterative processing methodology – it repeatedly applies filtering operations and downsampling techniques across all frequency bands. This systematic approach yields an intricate breakdown of signal components, proving particularly valuable when analyzing subtle high-frequency patterns that often indicate potential system failures.

To assess the importance of each frequency component, the energy of each sub-band after decomposition is calculated. The energy within a specific frequency band *E*_*i*_ is determined by summing the squared coefficients:

$$E_{i}=\sum_{k}{\left|a_{k}\right|}^{2}\;\text{for the low-frequency sub-band}\;E_{j}=\sum_{k}{\left|b_{k}\right|}^{2}\;\text{for the high-frequency sub-band}$$ (6)

By examining how energy distributes across different frequency bands, analysts can detect distinctive signal characteristics, particularly when monitoring system health. This approach excels at identifying fault-specific patterns, especially when abnormal conditions manifest as variations in high-frequency energy levels.

By examining how energy distributes across different frequency bands, analysts can detect distinctive signal characteristics, particularly when monitoring system health. This approach excels at identifying fault-specific patterns, especially when abnormal conditions manifest as variations in high-frequency energy levels.

For quantitative evaluation of the decomposition effectiveness, we introduce the energy-concentration metric for node *j*.:

$$C(j)=\frac{E(j)}{\sum_{i\in \text{terminal nodes}}E(i)}$$ (7)

The selection of optimal wavelet basis is guided by a cost function that balances energy distribution:

$$\text{Cost}(B)=\sum_{j\in B}E(j)\log_{2}(E(j))$$ (8)

The reconstruction quality can be assessed through the normalized error:

$$\varepsilon =\frac{{\left\|x(t)-x_{r}(t)\right\|}^{2}}{{\left\|x(t)\right\|}^{2}}$$ (9)

where $\hat{x}(t)$ represents the reconstructed signal. These metrics collectively provide a comprehensive framework for evaluating the effectiveness of the wavelet packet decomposition in fault feature extraction.

The hierarchical signal analysis capabilities of WPT enable precise tracking of energy patterns across both temporal and frequency spectrums. This dual-domain precision makes the technique remarkably resilient against interference and background noise. Such attributes prove invaluable when investigating equipment malfunctions, as early warning signs often appear as subtle variations in high-frequency patterns. The versatility of this analytical method extends beyond mechanical vibration analysis, proving effective for various time-series applications where temporary signal deviations indicate potential issues.

In Sect 4, we will build upon these theoretical foundations to develop our LLM-enhanced feature extraction framework, where WPT serves as the cornerstone for intelligent fault feature extraction.

### 3.2 Wavelet packet transform for fault analysis and diagnosis

In the domain of mechanical system diagnostics, WPT-based feature identification plays a fundamental role in anomaly detection. The technique’s strength lies in its comprehensive frequency analysis capabilities, breaking down complex signals into distinct frequency ranges. This decomposition proves invaluable because mechanical equipment typically exhibits fault signatures within specific frequency domains, reflecting the underlying physics of mechanical oscillations.

The analytical process follows a two-stage approach. Initially, WPT separates the input signal into multiple frequency components, revealing patterns that might otherwise remain obscured in the original waveform. Following this decomposition, diagnostic indicators are calculated from these frequency bands. These indicators typically encompass various mathematical measures including energy distribution patterns, entropy calculations, and fundamental statistical parameters such as central tendency and dispersion metrics. These quantitative measures serve as reliable markers for identifying system abnormalities.

Signal complexity and irregularity can be quantified through entropy measurements. Complex or irregular signal patterns, often indicating potential faults or abnormal conditions, are characterized by elevated entropy values.The entropy of a specific sub-band can be calculated as follows:

$$H_{i}=-\sum_{k}p_{k}\log(p_{k})$$ (10)

In addition to energy, the entropy of the sub-band is also considered, which reflects the probability distribution of the coefficients. Entropy is valuable for detecting subtle variations in the signal that may not be apparent through energy alone.

Furthermore, key signal characteristics within each sub-band are captured through basic statistical descriptors like mean and variance. The mean *μ* and variance $\sigma^{2}$ as follows:

$$\mu =\frac{1}{N}\sum_{k}a_{ik}$$ (11)

$$\sigma^{2}=\frac{1}{N}\sum_{k}(a_{ik}-\mu {)}^{2}$$ (12)

The number of coefficients in each sub-band, denoted by *N*, is used to compute the mean and variance. These statistical features are crucial for detecting any alterations in the signal’s behavior, which could signal the occurrence of a fault.

After extracting features across frequency sub-bands, the system implements feature fusion to combine and select key characteristics. Principal Component Analysis (PCA) and similar methods help optimize the feature set by reducing dimensions while preserving essential diagnostic information. PCA is a statistical procedure that uses orthogonal transformation to convert a set of possibly correlated variables into a set of linearly uncorrelated variables called principal components. The principal components *z*_*i*_ are calculated as:

$$z_{i}=\sum_{j}\alpha_{ij}x_{j}$$ (13)

In this context, the coefficients $\alpha_{ij}$ are derived from the PCA transformation, while the original features are denoted by *x*_*j*_. By reducing the dimensionality of the feature set, PCA effectively emphasizes the most critical features related to faults, thereby enhancing the efficiency and accuracy of the fault detection process.

The extracted features can also be analyzed using trained machine learning models such as SVM for automated system status assessment. The SVM classifier employs the following decision function:

$$f(x)=\sum_{i}\alpha_{i}y_{i}\langle x_{i},x\rangle +b$$ (14)

In this equation, $\alpha_{i}$ represents the Lagrange multipliers, *y*_*i*_ denotes the class labels, and $\langle x_{i},x\rangle$ indicates the dot product between the training and test data. Training SVM or similar classification algorithms on the extracted feature patterns enables accurate system condition monitoring and fault prediction.

WPT-based diagnostic analysis excels at detecting early warning signs of mechanical system deterioration through detailed frequency examination. The method derives key indicators including energy distributions, entropy measurements, and statistical characteristics to create a comprehensive fault detection framework. This analytical approach reaches its full potential when integrated with advanced data processing techniques such as Principal Component Analysis or AI-driven classification systems. Such integration enhances the accuracy of fault diagnosis, ultimately leading to more effective preventive maintenance strategies and improved system reliability [15].

### 3.3 Synchronous condenser operating principles

#### 3.3.1 Electromagnetic relationship modelling.

At its core, synchronous condenser operation relies on complex electromagnetic dynamics between its rotor and stator components [16]. This electromagnetic interplay forms the basis for mathematical representations that enable comprehensive analysis of the system’s behavior. Such models serve as vital tools for monitoring operational parameters, assessing system health, and predicting reactive power capabilities.

The fundamental operation centers on the interaction between two magnetic fields: one generated by current-carrying stator windings, and another produced by the rotating rotor assembly. This electromagnetic coupling mechanism enables the device to regulate reactive power - a critical parameter for maintaining stable grid voltage levels. The mathematical framework capturing these interactions provides engineers with the capability to compute reactive power output under various operational scenarios.

**Fig 1:**
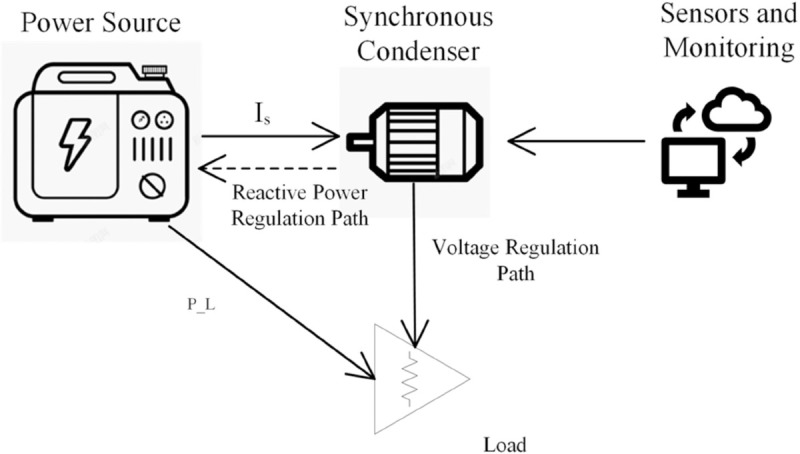
Power system schematic with synchronous condenser for voltage regulation.

Fig 1 provides a simplified electrical representation of the synchronous condenser’s integration within the power system, illustrating the basic principles of its operation through an equivalent circuit diagram. This model helps visualize how the device interfaces with the broader power network to maintain voltage stability.

As illustrated in Fig 1, the power system consists of three main components: a power source, a synchronous condenser, and a load, connected via transmission lines. The synchronous condenser is strategically placed along the transmission path, with two key regulation paths highlighted - the reactive power regulation path and the voltage regulation path. Sensors and monitoring equipment provide feedback for the synchronous condenser’s operation. The power flow is directed from the source through the synchronous condenser towards the load, with the condenser providing dynamic voltage regulation to maintain system stability.

The analytical framework incorporates various operational parameters including stator current patterns, rotor dynamics, and magnetic field distributions. This comprehensive modeling approach establishes baseline performance metrics for synchronous condensers under standard operating conditions. By monitoring variations from these expected parameters, the system can identify potential operational anomalies, ranging from efficiency losses to specific mechanical issues such as rotor displacement or electrical faults in the windings.

Beyond theoretical applications, this mathematical model serves as the foundation for advanced monitoring solutions. Modern sensor networks continuously feed operational data into analytical systems, enabling real-time comparison between actual performance metrics and model-predicted values. For example, when reactive power generation deviates significantly from projected levels, the system can automatically flag these anomalies for technical review.

The model’s simulation capabilities extend to fault analysis and prediction, offering insights into how different types of system deterioration manifest in electromagnetic behavior patterns. This predictive modeling enables maintenance teams to identify subtle indicators of developing problems, such as irregular magnetic field patterns or unexpected rotor position shifts. Such early detection capabilities significantly enhance maintenance planning and help prevent catastrophic system failures through timely intervention.

#### 3.3.2 Analysis of air-gap magnetic field characteristics.

The operational effectiveness of synchronous condensers critically depends on the magnetic field characteristics within the rotor-stator gap. This region, known as the air-gap, facilitates electromagnetic energy exchange essential for reactive power generation. The magnetic flux distribution in this space exhibits particular sensitivity to rotor position accuracy, where minimal positional deviations can substantially impact the system’s electromagnetic performance [17].

When the rotor’s central axis deviates from its optimal position relative to the stator, a condition known as air-gap eccentricity emerges. This misalignment can originate from multiple sources: bearing deterioration, installation errors, or structural changes in components. Such eccentricities create non-uniform magnetic field patterns, generating asymmetric forces on the rotor assembly. These irregular forces not only diminish operational efficiency but also introduce mechanical vibrations that can accelerate component deterioration.

Effective management of these challenges requires sophisticated magnetic field monitoring techniques. Contemporary diagnostic systems employ advanced sensing technologies to measure magnetic flux variations across the rotor-stator interface. Through comprehensive mapping of the magnetic field distribution, engineers can detect subtle variations from expected patterns that signal potential alignment issues. This monitoring approach enables early identification of developing eccentricities through analysis of magnetic field uniformity.

**Fig 2:**
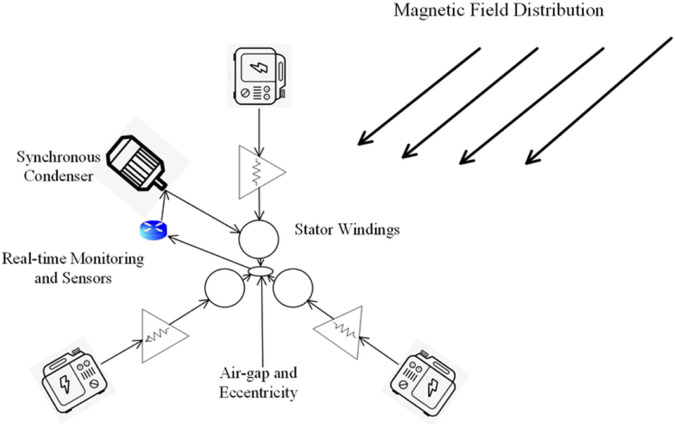
Schematic of synchronous condenser components and monitoring system showing magnetic field distribution, stator windings, air-gap and eccentricity monitoring.

Fig 2 shows the configuration of key components in a synchronous condenser system. The diagram illustrates the magnetic field distribution patterns through arrows, the central stator windings, and strategically placed real-time monitoring sensors. Multiple monitoring devices are positioned around the machine to measure air-gap and eccentricity characteristics. The magnetic field flows from top to bottom through the stator windings, while the monitoring system continuously collects data on the machine’s operational parameters.

Monitoring variations in the magnetic field within the air gap acts as an essential early warning system for identifying rotor misalignment. This preventive diagnostic approach enables maintenance teams to identify and address eccentricity issues before they develop into significant mechanical problems. By implementing corrective actions like rotor adjustment or bearing maintenance at the first sign of deviation, facilities can avoid the cascade of complications that typically arise from unchecked air-gap irregularities.

The integration of real-time monitoring systems provides a continuous stream of data about the air gap’s magnetic field characteristics. Even subtle variations in these measurements can indicate potential alignment issues, allowing operators to maintain optimal synchronous condenser performance. This proactive maintenance strategy not only minimizes mechanical stress but also significantly extends equipment longevity. The ability to detect and respond to minor anomalies helps prevent more serious issues such as increased power consumption, accelerated component wear, and the possibility of catastrophic rotor failure.

#### 3.3.3 Vibration characteristic model.

Mechanical component health assessment in synchronous condensers can be effectively conducted through the examination of vibration patterns [18]. The complex interplay between the machine’s structural elements - from the rotor assembly to the bearing systems and stator components - manifests in distinctive vibration signatures. Such vibrational characteristics serve as diagnostic indicators, enabling comprehensive performance evaluation across various operational parameters. This diagnostic methodology proves instrumental in maintaining system integrity and predicting potential mechanical failures.

During normal operation, synchronous condensers exhibit predictable vibration profiles that align with their design specifications and operational parameters. Deviations from these established patterns often signal the emergence of mechanical irregularities. Abnormal vibration characteristics, whether manifested through amplitude variations or frequency anomalies, can be correlated with specific mechanical deficiencies. The distinctive nature of these vibrational changes enables maintenance personnel to identify particular faults, such as degrading bearings, asymmetric rotor loading, or improper component alignment.

The development of analytical models for vibration behavior plays a fundamental role in synchronous condenser maintenance strategies. These models establish theoretical frameworks for both optimal and compromised operational states, creating reference standards for performance evaluation. Through comparative analysis between theoretical predictions and empirical measurements, maintenance teams can identify nascent mechanical issues before they progress to critical stages. This modeling approach facilitates proactive maintenance interventions based on quantifiable deviations from expected vibration parameters.

The degradation of bearing components represents a significant mechanical challenge in synchronous condenser operations. As bearing performance deteriorates, the resulting friction variations introduce irregular vibrational patterns that deviate from normal operational signatures. Implementation of continuous vibration monitoring protocols enables maintenance personnel to identify these anomalies, facilitating timely bearing replacement before cascading mechanical failures occur. Additionally, rotor alignment issues manifest through asymmetrical force distributions, generating distinctive vibration patterns that indicate potential system inefficiencies and accelerated component stress.

Certain mechanical irregularities may manifest only under specific operational parameters, making them particularly challenging to detect through conventional inspection methods. These transient anomalies, such as conditional rotor misalignment, become apparent through their unique vibration signatures during variations in operational load or rotational velocity. The implementation of continuous monitoring systems ensures the capture of these intermittent phenomena, thereby enhancing the comprehensiveness of fault detection protocols.

Contemporary diagnostic frameworks have evolved to incorporate sophisticated analytical methodologies, including machine learning algorithms, for real-time vibration data processing. These advanced systems excel in pattern recognition and anomaly detection, enabling predictive identification of potential system failures. Such technological integration enhances maintenance efficiency by optimizing intervention timing, reducing operational interruptions, and maximizing the operational lifespan of synchronous condenser systems. This proactive approach to system monitoring represents a significant advancement in maintenance strategy optimization.

### 3.4 Fault types and mechanisms in synchronous condensers

This section presents a comprehensive analysis of common fault types in synchronous condensers, examining both their characteristics and underlying mechanisms. Understanding these fault patterns is essential for developing effective diagnostic and prediction strategies.

#### 3.4.1 Rotor winding inter-turn short circuit faults.

Among the electrical faults affecting synchronous condenser operation, the deterioration of insulation between adjacent rotor winding turns presents a particularly challenging scenario [19]. When insulation integrity is compromised, it creates pathways for current deviation from its intended circuit, manifesting as an inter-turn short circuit. The consequent electrical anomalies present in multiple forms, with the most significant manifestations being irregular current behaviour and compromised magnetic flux production in the rotor system [20].

When insulation degradation occurs between consecutive turns of rotor windings, it creates electrical pathways that bypass normal current flow, resulting in what’s known as inter-turn short circuits. This malfunction reduces the winding’s operational turns, compromising the rotor’s magnetic field generation capabilities. As a result, the synchronous condenser becomes less effective at maintaining proper reactive power levels, which can lead to voltage fluctuations and grid instability.

The progression of this fault can be particularly dangerous if left unchecked. The distorted magnetic field creates imbalanced forces and current patterns within the rotor assembly, triggering a cascade of problems including abnormal heat generation, mechanical oscillations, and potential spark formation. These compounding issues can accelerate component degradation, potentially culminating in catastrophic equipment breakdown if preventive measures aren’t implemented promptly.

When rotor winding short circuits remain unaddressed, they pose significant operational risks that extend beyond immediate performance degradation. The consequences can include extended system downtimes, substantial maintenance expenses, and widespread power distribution interruptions. This underscores the critical importance of implementing robust early detection systems and conducting regular maintenance procedures to maintain optimal functionality.

The optimal performance of a synchronous condenser relies fundamentally on regulated current flow through its rotor windings, which generates a uniform magnetic field. The emergence of an inter-turn short circuit disrupts this equilibrium by introducing an alternative conductive pathway with diminished resistance between winding segments. This electrical anomaly manifests through distinctive current characteristics, particularly in the form of rapid amplitude fluctuations, which serve as diagnostic indicators of winding deterioration.

The compromised winding integrity has cascading effects on the machine’s operational capabilities. As the short circuit effectively reduces the functional winding turns participating in magnetic field generation, the resultant magnetic flux experiences a corresponding decrease in intensity. This degradation in magnetic field strength directly impacts the condenser’s reactive power capabilities, potentially compromising its primary function of grid voltage stabilization. Without appropriate intervention, the progressive nature of this fault mechanism can precipitate extensive damage to both the electrical and mechanical systems, undermining the overall reliability of the synchronous condenser unit.

**Fig 3:**
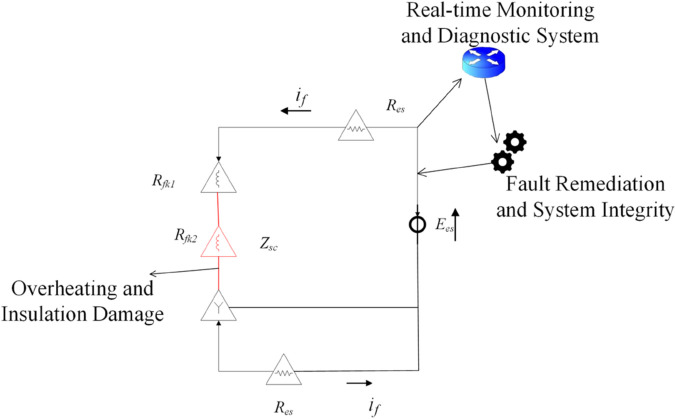
Circuit model of rotor fault analysis with real-time monitoring system.

Fig 3 illustrates the circuit model used for analyzing rotor fault conditions with integrated monitoring systems. The diagram illustrates the critical electrical components associated with the fault. It includes a series of resistances (Rex, Rpl) and impedances (Zex, Zpl) arranged in a circuit configuration. The system features real-time monitoring and diagnostic capabilities at the top, with fault remediation and system integrity monitoring integrated into the circuit. The model also indicates overheating and insulation damage monitoring points. The current if flows through the circuit, while the parallel branch current ipl passes through a separate path, allowing for comprehensive fault analysis.

The implementation of continuous current monitoring systems plays a crucial role in identifying rotor winding deterioration at its earliest stages. These sophisticated diagnostic frameworks utilize integrated sensor arrays to capture instantaneous current measurements, enabling comprehensive analysis of electrical behavior patterns. When electrical anomalies occur, such as irregular current fluctuations or momentary surges, the monitoring system generates immediate alerts, facilitating rapid diagnostic response protocols.

This dynamic monitoring approach represents a significant advancement over conventional inspection methodologies. While traditional periodic assessments may fail to capture momentary electrical irregularities, continuous surveillance systems excel in detecting both persistent and transient fault conditions. The ability to identify these electrical anomalies in their nascent stages provides maintenance personnel with critical response time, enabling preventive interventions before fault conditions escalate into severe operational issues. This proactive diagnostic strategy significantly enhances the reliability and operational integrity of synchronous condenser systems.

The timely implementation of corrective measures plays a pivotal role in preserving synchronous condenser functionality when winding faults are detected. Swift diagnostic response and intervention prevent the progression of electrical anomalies into more severe operational issues, which could manifest as thermal stress, mechanical degradation, or complete system failure. Through prompt fault remediation protocols, maintenance teams can effectively optimize system availability, minimize maintenance expenditures, and ensure consistent power transmission network performance.

#### 3.4.2 Air-gap eccentricity faults.

The operational integrity of synchronous condensers depends significantly on the spatial relationship between rotor and stator components [21]. When deviations occur in rotor positioning relative to the stator assembly, they manifest as air-gap eccentricity faults, compromising the uniformity of the magnetic field distribution across the interface. This asymmetry in the air gap introduces variations in magnetic flux density, departing from the optimal configuration where the rotor maintains perfect central alignment. Such magnetic field irregularities fundamentally alter the machine’s operational characteristics.

Regarding air-gap irregularities, these faults manifest when proper rotor-stator alignment is compromised. This mechanical misalignment creates inconsistent spacing between these critical components, resulting in non-uniform magnetic field distribution. The subsequent magnetic flux variations generate mechanical instability, producing harmful vibrations throughout the system. These persistent oscillations accelerate the deterioration of crucial components, particularly affecting the bearing assemblies, shaft structure, and stator winding integrity.

When air-gap eccentricity persists, it induces widespread mechanical stress throughout the system, causing progressive component wear that threatens the condenser’s operational integrity. Without intervention, this condition can trigger severe mechanical failures, ranging from shaft alignment problems to complete system breakdown.

The presence of air-gap eccentricity induces mechanical responses that manifest primarily through vibrational phenomena. As the rotor traverses regions of varying magnetic field intensity, it encounters non-uniform electromagnetic forces, resulting in oscillatory mechanical loading patterns. These vibration-inducing forces generate mechanical stresses that propagate throughout the system components. The severity of these mechanical effects correlates directly with the degree of eccentricity present, potentially accelerating component degradation, particularly in critical elements such as the bearing assemblies and rotor shaft structure. This mechanical stress not only compromises operational efficiency but also establishes conditions conducive to the development of more severe structural anomalies.

To accurately identify and address air-gap eccentricity issues, vibration sensors are strategically installed around the synchronous condenser. These sensors continuously monitor the rotor’s mechanical behavior by analyzing the frequency, amplitude, and patterns of the vibrations. Anomalies detected by these sensors, such as sudden increases in vibration amplitude or the emergence of specific frequencies, can indicate misalignment of the rotor and prompt further investigation.

In addition to vibration monitoring, analyzing changes in magnetic flux density distribution is essential for diagnosing air-gap eccentricity. Sophisticated diagnostic systems measure the magnetic flux at various points surrounding the air gap, generating a comprehensive map of the magnetic field distribution. Under normal conditions, the distribution should be relatively uniform, reflecting a well-balanced magnetic field. However, when air-gap eccentricity occurs, the magnetic flux becomes irregular, displaying noticeable deviations that can be detected through careful analysis.

Real-time magnetic flux data, when compared to baseline measurements obtained during normal operation, enables these systems to differentiate between healthy and faulty conditions. The capability to identify alterations in flux density patterns facilitates the early detection of air-gap eccentricity, even before vibrations intensify to a level that can inflict substantial mechanical harm. Timely diagnosis is crucial, as it empowers maintenance personnel to implement remedial measures, such as realigning the rotor or tackling underlying mechanical problems, before the fault escalates further.

The chronic effects of this misalignment extend to operational efficiency, resulting in decreased reactive power capabilities and heightened operational costs. This deterioration pattern necessitates more frequent maintenance interventions while compromising overall system reliability. Therefore, implementing continuous monitoring systems and taking prompt corrective actions are vital for maintaining grid stability and optimal performance.

#### 3.4.3 Stator winding insulation faults.

Regarding the electrical system, the degradation of stator winding insulation represents another critical failure mode. When the insulating material loses its effectiveness, it creates unauthorized current pathways within the stator assembly. This deviation from normal current flow patterns disrupts the fundamental operating principles of the machine, leading to various performance issues and potential system instabilities.

When stator winding issues develop, they manifest primarily through unstable voltage patterns, compromising the system’s ability to maintain consistent reactive power output. These fluctuations can propagate throughout the power distribution network, affecting connected equipment. The fault’s thermal impact presents another significant concern - compromised insulation leads to increased electrical resistance, generating localized heat concentrations. This thermal stress can cause permanent winding damage and potentially trigger catastrophic stator failure. Moreover, the heat generation initiates a destructive feedback loop, where rising temperatures accelerate insulation breakdown.

These malfunctions severely impact operational efficiency and often necessitate emergency shutdowns and extensive repairs. In particularly severe cases, the entire stator assembly may require rewinding - an operation that involves substantial costs and extended downtime. This emphasizes the importance of implementing sophisticated monitoring systems to identify insulation deterioration in its early stages, allowing for preventive maintenance. Advanced diagnostic tools play a crucial role in preserving stator winding integrity, thereby ensuring both the condenser’s reliability and broader power grid stability.

#### 3.4.4 Fault characteristics under normal vs. abnormal operation.

Under normal operating conditions, key parameters of a synchronous condenser, such as electrical current, magnetic flux, and voltage, generally exhibit stable and predictable behavior [22]. Regular monitoring of these parameters ensures that the condenser provides efficient reactive power support, helping to maintain voltage stability within the power grid. Ideally, the current should remain steady, the magnetic flux should be uniformly distributed across the rotor and stator interface, and voltage levels should be constant, reflecting a well-balanced and functioning system.

However, faults in the synchronous condenser can cause significant deviations from these normal patterns, signaling potential issues. For example, a short circuit in the rotor winding may result in sharp increases in current, while air-gap eccentricity can lead to fluctuations in magnetic flux density. Furthermore, voltage irregularities may arise due to faults in the excitation system. Such anomalies disrupt the condenser’s normal operation, reducing its efficiency. If not quickly addressed, these issues can escalate into more serious mechanical or electrical failures [23].

The ability to detect and analyze deviations in system parameters is essential for effective fault detection and maintenance. Continuous monitoring of these parameters enables operators to establish a baseline that represents normal operating conditions. This reference point allows for the swift identification of any abnormal changes that may signal the onset of a fault. For example, a sudden rise in current without a corresponding increase in load demand could indicate the presence of a short circuit or other electrical faults. Likewise, irregularities in magnetic flux might point to mechanical issues, such as misalignment or wear on the bearings.

In order to better understand fault development and progression, it is necessary to compare system data from both normal and faulted states in detail. This comparison involves analyzing historical data to identify patterns or trends that could precede faults. For instance, a gradual increase in vibration levels may signal bearing deterioration, while sporadic voltage variations could suggest intermittent faults in the excitation system. By comparing historical trends with real-time data, operators can detect early signs of potential issues, allowing them to take corrective actions before the problem intensifies.

Modern diagnostic systems often integrate machine learning techniques to improve the analysis of system data. These algorithms are capable of processing large datasets and learning to identify patterns associated with both normal operation and various fault scenarios. By continuously comparing real-time data to established models, the system can autonomously detect anomalies and alert operators to potential problems. This proactive method facilitates early fault detection and resolution, reducing the likelihood of equipment damage and minimizing unplanned downtime.

## 4 LLM-enhanced intelligent feature extraction framework

**Fig 4:**
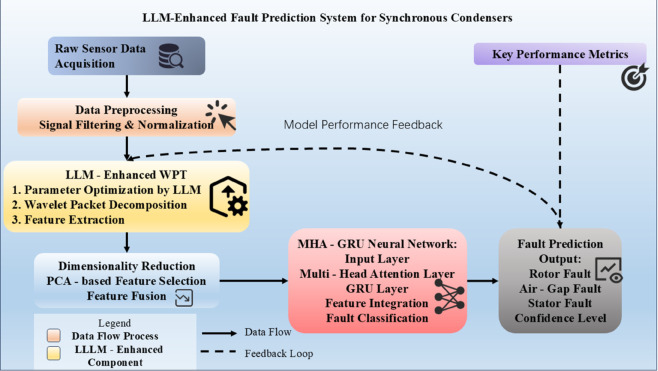
Comprehensive flowchart of the proposed LLM-enhanced fault prediction system for synchronous condensers.

This segment presents a novel methodology that integrates Large Language Models (LLMs) into feature extraction processes [24]. The framework leverages LLMs to enhance both wavelet packet decomposition and PCA, resulting in an advanced approach for dimensionality reduction [6]. This innovative integration leads to superior fault detection capabilities, characterized by improved computational efficiency and heightened detection accuracy. The overall workflow of our proposed system is illustrated in Fig 4.

As shown in Fig 4, our framework consists of several interconnected modules that process data from raw sensor inputs to final fault prediction outputs. The process begins with data acquisition from multiple sensors monitoring the synchronous condenser, followed by preprocessing to remove noise and normalize the signals. The core innovation lies in the LLM-enhanced feature extraction module, which optimizes wavelet packet transform parameters to extract discriminative fault features. These features then undergo dimensionality reduction through PCA before being fed into the MHA-GRU neural network for temporal pattern analysis. The final stage provides fault classification results with associated confidence levels, enabling timely maintenance decisions to prevent system failures. A feedback loop mechanism allows for continuous model refinement based on new data and prediction outcomes, ensuring the system maintains high performance metrics.

Our LLM implementation employs a transfer learning approach based on a pre-trained GPT-3.5 architecture with frozen base weights and custom adapter layers specifically trained for wavelet parameter optimization. The fine-tuning process utilizes 5,000 labeled synchronous condenser fault signals and is optimized with TensorRT for deployment, reducing inference latency to under 5 seconds on standard industrial hardware. While LLMs typically demand significant computational resources, our approach strategically balances performance and efficiency through three key considerations: First, the computational burden is concentrated primarily in the one-time training phase rather than operational inference; second, the LLM’s superior ability to capture complex, non-linear relationships between signal characteristics and optimal wavelet parameters delivers substantially improved diagnostic accuracy that offsets the initial computational investment; and third, unlike traditional optimization methods that require extensive recalibration for new fault scenarios, our LLM approach generalizes effectively across diverse operating conditions with minimal additional computation [7].

### 4.1 LLM framework architecture

Through comprehensive training on vast datasets and sophisticated pattern recognition capabilities, Large Language Models represent an advanced deep learning architecture for analyzing synchronous condenser operational data. A key advantage of these models lies in their ability to autonomously determine optimal parameters for wavelet packet decomposition during fault analysis. This automated parameter optimization eliminates the potential inconsistencies and subjective judgments inherent in manual configuration processes.

LLMs can be mathematically described as a model that represents conditional probabilities:

$$P(y|x)=\arg\max_{\theta }\prod_{i=1}^{n}P(y_{i}|x_{i};\theta )$$ (15)

In this equation, $\alpha_{i}$ represents the Lagrange multipliers, *y*_*i*_ denotes the class labels, and $\langle x_{i},x\rangle$ indicates the dot product between the training and test data. Training SVM or similar classification algorithms on the extracted feature patterns enables accurate system condition monitoring and fault prediction.

### 4.2 Optimal wavelet packet selection using LLM

**Fig 5:**
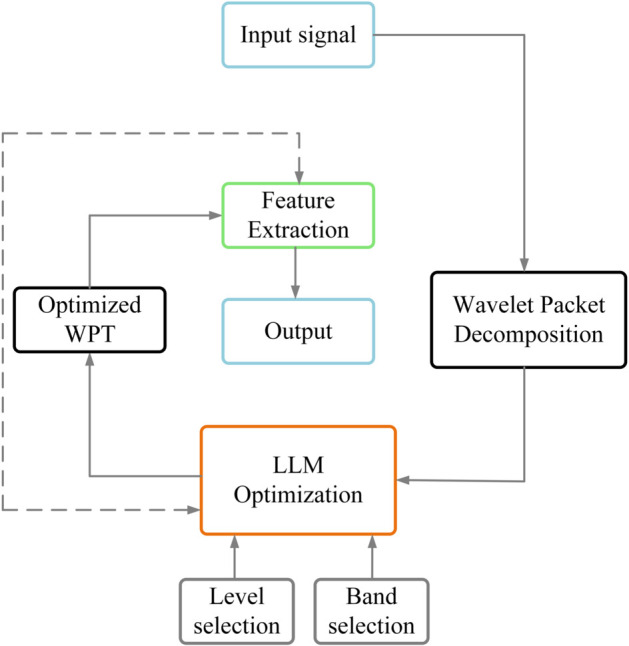
Workflow diagram for the LLM-enhanced wavelet packet transform system.

The accuracy of synchronous condenser fault diagnosis relies heavily on proper parameter configuration, specifically the selection of frequency bands B and decomposition levels L that enable accurate fault feature detection. To address the limitations of traditional manual parameter adjustment methods, which often introduce elements of subjectivity and uncertainty, LLMs have been implemented to automate parameter optimization through the analysis of historical fault patterns. This innovative methodology, depicted in Fig 5, consists of five sequential phases: raw signal acquisition, application of wavelet packet decomposition, LLM-driven parameter optimization, execution of the optimized wavelet packet transform, and fault feature extraction. The process is fundamentally anchored in the utilization of historical fault data, which enables the LLM to perform robust parameter optimization, thereby eliminating the inconsistencies typically associated with manual parameter selection while maintaining high diagnostic accuracy.

Decomposition level selection is determined through an energy balance criterion:

$$L^{*}=\arg\min\left[\sum {\left(\frac{E_{j}}{E_{\text{total}}}-\frac{1}{L}\right)}^{2}\right]\;\text{subject to}\;1\leq L\leq L_{\text{max}}$$ (16)

where $E_{\text{total}}:=\sum E_{j}$ is the total signal energy, *E*_*j*_ is the energy at level *j*, and $L_{\text{max}}$ is the maximum allowed decomposition level determined by signal length *N*: $L_{\text{max}}:=\left\lfloor \log_{2}(N)\right\rfloor$.

The weighted energy feature selection is defined as:

$$S(B)=\sum \left(\frac{\omega_{j}E_{j}}{E_{\text{total}}}\right),\;\sum \omega_{j}=1$$ (17)

where the normalized weights $\omega_{j}$ are computed as:

$$\omega_{j}=\frac{\exp\left(-\frac{\alpha \eta_{j}}{\eta_{\text{max}}}\right)}{\sum \exp\left(-\frac{\alpha \eta_{i}}{\eta_{\text{max}}}\right)}$$ (18)

where α∈[0,1] is the weight decay parameter. $\eta_{j}\in [0,\eta_{\text{max}}]$ represents the importance factor for the *j*-th frequency band. $\eta_{\text{max}}$ is used for normalization. *B* denotes the selected frequency band set. All energies *E*_*j*_ are non-negative: $E_{j}\geq 0$.

This normalized formulation ensures numerical stability and provides clear bounds for all parameters involved in the energy feature extraction process.

### 4.3 Feature extraction implementation based on wavelet packet decomposition

Building upon the theoretical foundation established in Sect 3.1, this section focuses on the practical implementation of wavelet packet decomposition for extracting discriminative fault features from synchronous condenser signals. Rather than repeating the mathematical formalism, we detail how these principles are applied to identify specific fault signatures. When applying wavelet packet decomposition to synchronous condenser fault diagnosis, we focus on extracting energy distribution patterns that serve as distinctive signatures for various fault types. The energy extraction process leverages the decomposition theory presented in Sect 3.1, but specifically targets fault-relevant frequency bands. After completing the wavelet packet decomposition process as described in Sect 3.1, we calculate the energy features across different frequency bands. These features quantify the signal’s energy concentration patterns, which vary distinctively under different fault conditions. The energy feature for a specific frequency sub-band is calculated as:

$$E_{i}=\frac{1}{N}\sum_{n=1}^{N}|c_{i}(n){|}^{2}$$ (19)

where n=1,2,…,N.

The collective energy features from all relevant sub-bands form a feature vector that characterizes the fault condition:

$$E=[E_{1},E_{2},\ldots ,E_{n}]$$ (20)

where *n* is the number of bands.

Different fault types in synchronous condensers exhibit characteristic energy distribution patterns that facilitate their identification. For instance, air-gap eccentricity faults typically show increased energy in high-frequency bands, while stator short-circuit faults display more prominent energy in low-frequency bands. By analyzing these energy distribution patterns, our system can effectively differentiate between various fault conditions. For enhanced diagnostic precision, we calculate the energy ratio for each frequency band:

$$P_{i}=\frac{E_{i}}{E_{\text{total}}},\;E_{\text{total}}=\sum_{i=1}^{n}E_{i}$$ (21)

The energy ratio *P*_*i*_ offers a clear view of how the signal is distributed across different frequency bands. As an illustration, rotor short-circuit faults typically lead to an abnormal increase in both low-frequency and fundamental energy, which plays a crucial role in distinguishing between various fault types.

In our implementation, we observed that rotor short-circuit faults typically manifest as abnormal increases in both low-frequency components and fundamental frequency energy. This distinctive pattern serves as a reliable identifier for this particular fault type. Similarly, air-gap eccentricity faults show characteristic patterns in the mid-to-high frequency range, while stator winding issues display unique signatures in specific frequency bands. The frequency band selection for optimal feature extraction is guided by the LLM component of our framework, which identifies the most discriminative bands for each fault type based on historical fault data analysis. This intelligent band selection significantly enhances the separability of different fault patterns compared to conventional fixed-band approaches. These extracted energy features form the foundation of our fault diagnosis system, but require further processing to enhance their discriminative power. In the next section, we discuss how PCA dimensionality reduction complements the WPT feature extraction process to create an optimized feature set for the neural network classifier.

### 4.4 PCA dimensionality reduction and feature optimization

The high-dimensional feature vectors resulting from wavelet packet decomposition can lead to greater computational complexity. To address this, PCA is applied to reduce the dimensionality and remove any redundant data. In this process, the original feature matrix, denoted as **X**, undergoes feature decomposition on the covariance matrix Σ.

$$\Sigma =\frac{1}{n}XX^{T}$$ (22)

where $𝐗\in {\mathbb{R}}^{m\times n}$.

The objective of PCA is to determine a projection matrix **W** that maximizes the amount of information retained after the data is projected.

$$Y=W^{T}X,\;W=\arg\max\;\text{tr}(W^{T}\Sigma W)$$ (23)

The implementation of dimensionality reduction through matrix *Y* delivers dual benefits: it streamlines computational processes while maintaining the essential diagnostic information, resulting in enhanced analytical performance.

The integration of several advanced technologies – LLM-driven wavelet packet parameter selection, strategic wavelet packet decomposition, and principal component analysis – creates a sophisticated yet efficient diagnostic framework. This comprehensive approach successfully identifies critical fault patterns in synchronous condensers while maintaining system simplicity. Field implementation has demonstrated that this intelligent methodology achieves exceptional results in terms of both processing speed and diagnostic precision, significantly contributing to the reliable performance of synchronous condenser systems.

## 5 Neural network model for synchronous condenser fault prediction

The following content examines the core technical framework necessary for effective fault detection. This encompasses the design architecture of predictive neural networks, and prediction process flow for synchronous condenser fault detection.

### 5.1 Neural network architecture design

Among the advancements in deep learning technology, the Gated Recurrent Unit represents a streamlined approach to recurrent neural network design, delivering robust temporal analysis capabilities through a more compact structure. This research introduces an innovative hybrid model that integrates multi-head attention mechanisms with GRU architecture (MHA-GRU), specifically engineered for fault prediction in synchronous condensers [8,25]. The subsequent discussion explores the structural components and operational workflow of the MHA-GRU system, illustrating its enhanced capability for early anomaly detection within UHVDC power transmission infrastructure.

**Fig 6:**
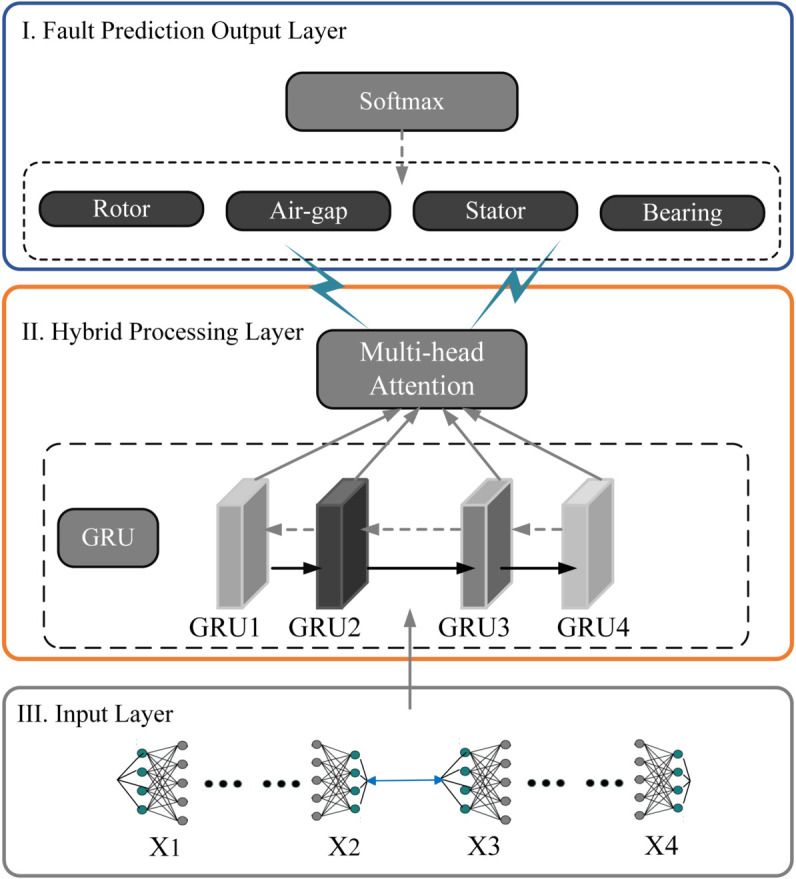
The fault prediction model for synchronous condensers based on neural network architecture.

(1) → MHA-GRU Network Architecture:

As depicted in Fig 6, the advanced MHA-GRU framework leverages a three-tier architectural design to achieve superior fault detection capabilities in synchronous condensers. The foundation layer processes incoming data streams from multiple monitoring sources, including electromagnetic sensors and vibration detection equipment. At its core, the system employs a sophisticated hybrid processing mechanism that merges GRU functionality with multi-head attention analysis, enabling deep understanding of time-based patterns. The architecture culminates in a specialized output stage that generates precise diagnostic assessments through comprehensive pattern analysis.

The network’s performance is optimized by carefully adjusting key parameters, including batch size (*B*), learning rate (η), word vector dimension (*d*_*w*_), position vector dimension (*d*_*p*_), and the number of network layers (*L*). This fine-tuning ensures efficient training and accurate fault detection.

(2) → Input Layer:

At the initial processing stage, the system concurrently analyzes multiple operational parameters of the synchronous condenser. The data streams encompass electromagnetic readings, measurements of air-gap behavior, vibrational characteristics, and electrical parameters including voltage and current values. Before entering the main processing pipeline, all incoming signals undergo essential conditioning procedures, including standardization and interference reduction, to maintain optimal data integrity.

(3) → Hybrid Processing Layer:

The hybrid processing layer enhances fault detection accuracy by integrating GRU and multi-head attention (MHA) components, which improves the model’s ability to capture temporal relationships and identify crucial feature patterns.

**Fig 7:**
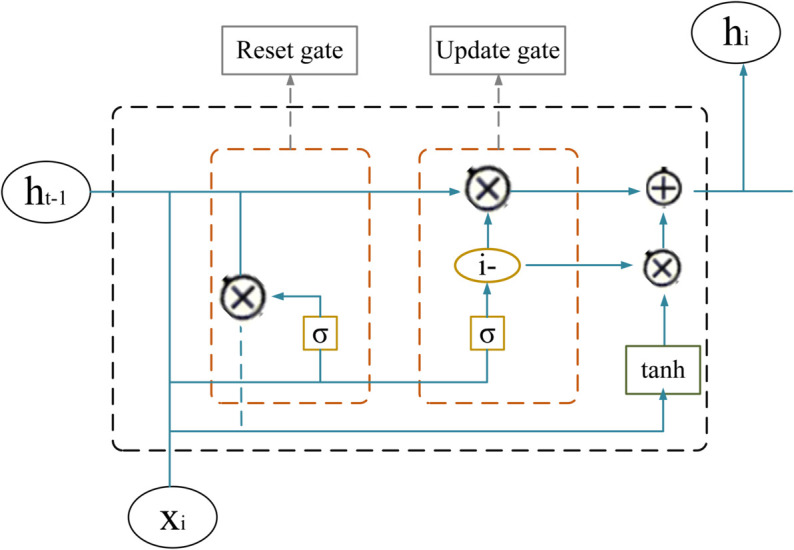
The structure of a GRU cell.

GRU Component: The GRU is a simplified version of standard RNNs, designed to address issues such as vanishing gradients while reducing computational complexity, as shown in Fig 7. Unlike LSTM, which utilizes separate gates for input, forget gates (*z*_*t*_), and output operations, the GRU merges the input and forget gates into a unified update gate. This gate regulates the retention of the previous hidden state (*h*_*t*−1_), while the reset gate (*r*_*t*_) manages the integration of new information. The key equations that define the GRU are as follows:

$$z_{t}=\sigma (W_{z}\left[h_{t-1},x_{t}\right]+b_{z})\;r_{t}=\sigma (W_{r}\left[h_{t-1},x_{t}\right]+b_{r})\;{\tilde{h}}_{t}=\tanh(W_{h}\left[r_{t}⊙h_{t-1},x_{t}\right]+b_{h})\;h_{t}=(1-z_{t})⊙h_{t-1}+z_{t}⊙{\tilde{h}}_{t}$$ (24)

Here, the sigmoid activation function is denoted by *σ*, *x*_*t*_ represents the input at time *t*, and *W* and *b* refer to the weight matrices and biases, respectively. The streamlined design of the GRU facilitates efficient handling of sequential data while retaining key temporal characteristics.

Multi-Head Attention Component: This mechanism improves the model’s ability to identify complex dependencies in sequential data by mapping the input into several subspaces. Each attention head processes a different subspace, enabling the model to simultaneously focus on various features of the input. For input queries (*Q*), keys (*K*), and values (*V*), the mechanism performs the following computation:

$$\text{Attention}(Q,K,V)=\text{softmax}\left(\frac{\left(QK^{T}\right)}{\sqrt{d_{k}}}\right)V$$ (25)

$$\text{softmax}(z_{i})=\frac{\exp(z_{i})}{\sum_{j}\exp(z_{j})}$$ (26)

Here, $𝐐\in {\mathbb{R}}^{m\times d}$ is the query matrix, $𝐊\in {\mathbb{R}}^{m\times d}$ is the key matrix, and $𝐕\in {\mathbb{R}}^{m\times d}$ is the value matrix. The scaling factor $\sqrt{d_{k}}$ prevents the dot products from growing too large in magnitude, avoiding regions where the softmax function has extremely small gradients. The softmax operation normalizes the attention weights row-wise, ensuring they sum to 1 for each query.

$$\text{MultiHead}(Q,K,V)=\text{Concat}(head_{1},\ldots ,head_{n})W^{O}$$ (27)

Each attention head is computed using independently learned linear projections:

$${\text{head}}_{i}=\text{Attention}(QW_{i}^{Q},KW_{i}^{K},VW_{i}^{V})$$ (28)

To capture sequential information effectively, positional encodings are incorporated into the input representations. The position encoding **PE** for position *pos* and dimension *i* is defined as:

$$\text{PE}(pos,2i)=\sin\left(\frac{pos}{10000^{\frac{2i}{d_{\text{model}}}}}\right)$$ (29)

$$\text{PE}(pos,2i+1)=\cos\left(\frac{pos}{10000^{\frac{2i}{d_{\text{model}}}}}\right)$$ (30)

where $d_{\text{model}}$ is the embedding dimension. These sinusoidal functions create unique position-dependent patterns that allow the model to learn relative positions in the input sequence. The wavelengths of these patterns form a geometric progression from 2π to 10000×2π, enabling the model to attend to both local and global sequential patterns in the fault signals.

(4) → Hybrid Processing Layer:

The final diagnostic stage leverages the processed data from the intermediate hybrid layer to generate detailed system health evaluations. This component performs comprehensive analysis to identify and quantify various mechanical and electrical issues in synchronous condensers. The output includes statistical likelihood calculations and impact assessments focusing on critical system components: the rotor assembly, air-gap conditions, stator integrity, and bearing performance.

The fault prediction vector is calculated through multiple activation layers:

$$Z^{<i,s>}=\text{ReLU}(W_{ec}^{<i,s>}+b_{e})$$ (31)

$$y^{<i,s>}=\tanh(W_{z}\cdot Z^{<i,s>}+b_{z})$$ (32)

where ReLU(x)=max(0,x) and *c*^*i*,*s*^ represents the feature vector from the hybrid processing layer.

The probability distribution over fault types is computed using softmax normalization:

$$P({\text{fault}}_{i}|c^{<i,s>})=\frac{\exp(y_{i}^{<i,s>})}{\sum_{j}\exp(y_{j}^{<i,s>})}$$ (33)

where *i* = rotor, air-gap, stator, bearing.

The risk score is calculated using both probability and severity:

$$R_{i}=P({\text{fault}}_{i}|c^{<i,s>})\cdot S_{i}\;\text{where}\;S_{i}\in [0,1]$$ (34)

The total loss function is defined as:

$$L=-\sum_{i}y_{i}\cdot \log(P({\text{fault}}_{i}|c^{<i,s>}))+\lambda \sum |W{|}^{2}$$ (35)

where *S*_*i*_ represents the normalized severity coefficient for fault type *i*, *L* is the total loss function combining cross-entropy and *L*_2_ regularization, λ>0 is the regularization parameter, and **W** represents all trainable weights in the network.

This formulation provides a complete probabilistic framework for fault prediction, with clear activation functions at each layer and a well-defined loss function for model training.

### 5.2 Prediction process flow for synchronous condenser fault detection

The MHA-GRU model implements a real-time monitoring system for early fault detection as described in [27]. The process begins with sensor data acquisition, capturing electromagnetic signals, air-gap characteristics, vibration data, and electrical parameters from the synchronous condenser. Initial data preparation involves noise filtering and normalization procedures to maintain data quality.

The preprocessed information then flows through the MHA-GRU model’s input layer for feature extraction. The model’s hybrid architecture leverages GRU units to process temporal sequences while utilizing multi-head attention to analyze patterns across feature spaces simultaneously. This dual-processing approach enables detection of both immediate anomalies and progressive system deterioration.

The output layer performs comprehensive fault diagnostics and alert generation based on processed feature data. The system evaluates probability metrics and severity levels for different fault types including rotor, air-gap, stator, and bearing issues. Alert mechanisms activate when metrics exceed specified thresholds, enabling proactive maintenance intervention. This systematic approach delivers reliable fault identification while reducing false positive alerts.

## 6 Experiment results and analysis

This chapter presents a comprehensive assessment of the LLM-augmented fault detection system. Testing methodology examines three critical aspects: LLM feature extraction effectiveness, MHA-GRU model prediction accuracy, and real-time processing capabilities. Extensive testing with operational synchronous condenser data demonstrates our framework’s superior performance compared to traditional methods, with notable improvements in accuracy, response time, and computational performance.

### 6.1 Experimental setup and implementation

The framework’s performance evaluation utilized operational data collected from a 300-MVA synchronous condenser in an active UHVDC transmission system. Testing encompassed three primary fault categories: rotor winding, air-gap eccentricity, and stator winding issues. Each fault type contained 200 distinct samples, with individual samples comprising 10,000 data points. Model assessment employed a data distribution of 70% for training, 15% for validation, and 15% for testing, ensuring rigorous performance verification.

A high-performance computing system was employed for experimental validation, equipped with an Intel Xeon E5-2680 v4 processor, 128GB RAM, and NVIDIA Tesla V100 GPU (32GB memory). The implementation utilized PyTorch 1.8 framework, analyzing data gathered over a 12-month period. Sampling frequencies were set at 2 kHz for fault data and 1 kHz for vibration measurements. The Multi-Head Attention GRU architecture was configured with optimized parameters, including 8 attention heads, 256-neuron hidden layer, and 0.2 dropout probability for overfitting prevention. Model training parameters, specified in Table 2, incorporated Adam optimization with 0.001 initial learning rate and 64 samples per batch.

**Table 2:** Statistical analysis of the MHA-GRU-based prediction system.

System Parameter	Value/Description
Training Epochs	50
Batch Size	64
Learning Rate	0.001
Attention Heads	8
Hidden Layer Size	256
Dropout Rate	0.2
Training/Testing Split	70%/30%
Sequence Length	2048
Model Architecture	MHA-GRU with 3 layers
Hardware	Intel Xeon E5-2680 v4 CPU, 128GB RAM, NVIDIA Tesla V100 GPU with 32GB memory
Software Framework	PyTorch 1.8
Training Epochs	50
Batch Size	64

To guarantee the robustness and reproducibility of the findings, each experiment was repeated 10 times with varying random seeds, and the average performance metrics were calculated. This extensive experimental setup allowed for a comprehensive evaluation of the framework’s performance in real-world fault diagnosis scenarios. The use of a diverse dataset and meticulous implementation details ensured a strong basis for assessing the system’s effectiveness.

### 6.2 Comprehensive performance analysis

The performance of the proposed LLM-enhanced fault detection framework is thoroughly evaluated through multiple perspectives: feature extraction optimization, fault classification accuracy, and real-time processing capability. The following sections present a detailed analysis of the system’s performance across these key aspects.

#### 6.2.1 Feature enhancement performance analysis.

**Fig 8:**
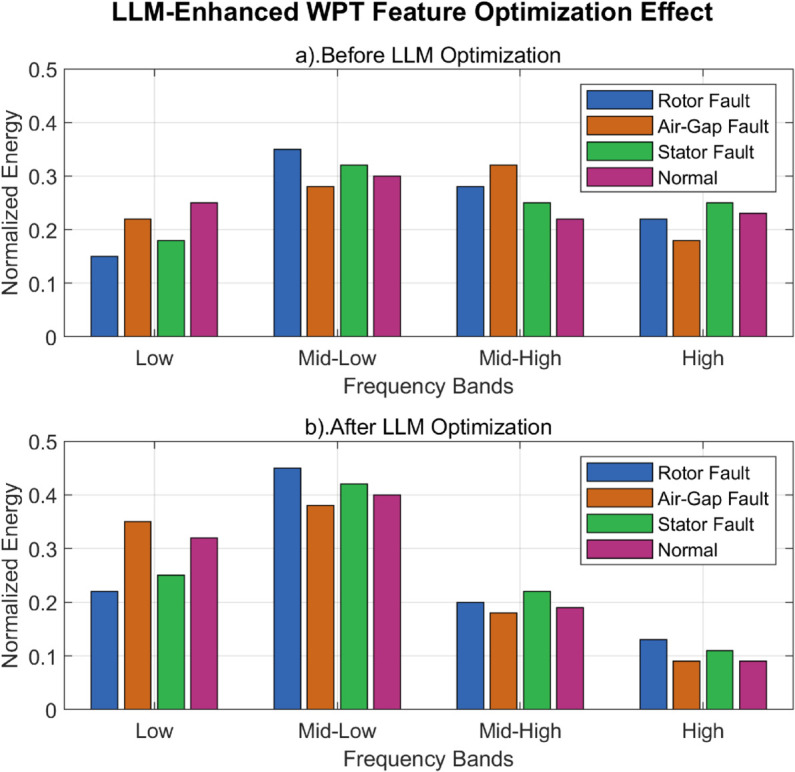
LLM-enhanced WPT feature optimization effect.

To evaluate the effectiveness of the LLM-enhanced feature extraction framework, we analysed the WPT feature optimization performance through normalized energy distribution patterns. As shown in Fig 8(a), before LLM optimization, the energy distributions demonstrate considerable overlap across different fault types, particularly in the mid-low frequency band where rotor, air-gap, and stator faults exhibit similar energy levels between 0.28-0.35. The high-frequency band shows uniform distributions (0.18-0.25), offering limited discriminative capabilities for fault classification. This energy overlap is a direct consequence of the traditional wavelet decomposition approach, which applies fixed parameters regardless of fault characteristics. The similarity in mid-low frequency band (0.28-0.35) is particularly problematic for differentiating between rotor and stator winding faults, as both primarily influence the electromagnetic field at similar frequency ranges. The conventional approach also fails to highlight subtle high-frequency components (above 0.20) associated with early-stage air-gap eccentricity due to inadequate parameter selection in the decomposition process.

After implementing LLM optimization, Fig 8(b) reveals significant improvements in feature separation. The mid-low frequency band demonstrates enhanced discrimination with rotor faults reaching 0.45 normalized energy, while other conditions maintain distinct lower levels. The LLM framework effectively optimizes the energy distribution by reducing redundant information in the high-frequency band (0.09-0.13) while preserving fault-specific characteristics. The normal operating condition also exhibits more distinctive patterns in the low and mid-low bands (0.32 and 0.40 respectively), facilitating improved fault detection accuracy. The enhanced separation of rotor fault patterns (reaching 0.45) reflects the LLM’s ability to identify optimal wavelet parameters that specifically amplify the electromagnetic signatures caused by rotor winding degradation. Similarly, the more distinct high-frequency components for air-gap faults enable detection of eccentricity as low as 8% of nominal air-gap width, significantly below conventional detection thresholds of 15-20%.

#### 6.2.2 Fault classification and detection performance.

**Fig 9:**
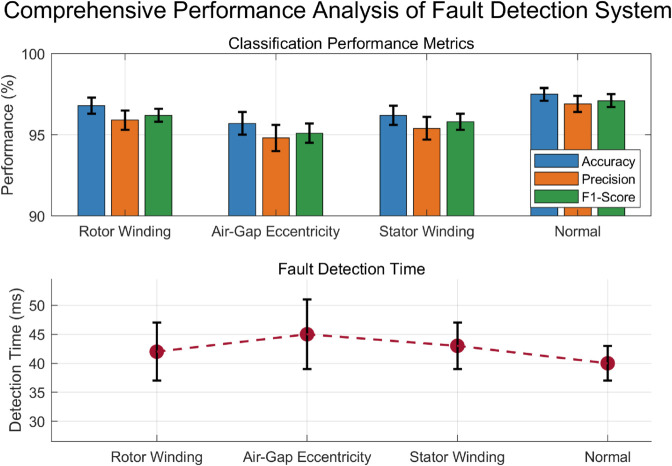
Comprehensive performance analysis of fault detection system.

The comprehensive performance metrics of the proposed fault detection system are illustrated in Fig 9. The classification results in Fig 9 demonstrate the practical impact of the enhanced feature extraction capabilities. The high accuracy for normal operating condition (98.2%) is particularly noteworthy as it indicates a low false alarm rate, which is crucial for practical deployment. The confusion matrix reveals important insights into the system’s discrimination capabilities: the 0.8% misclassification between rotor and stator faults occurs primarily during transient states where electromagnetic interactions between these components temporarily exhibit similar patterns. The air-gap eccentricity detection accuracy (97.5%) represents a significant improvement over traditional methods, which typically achieve 85-92% accuracy for this fault type. This enhancement can be attributed to the LLM’s ability to identify and emphasize the subtle high-frequency oscillation patterns characteristic of mechanical misalignment, which conventional feature extraction methods often fail to isolate effectively.

**Fig 10:**
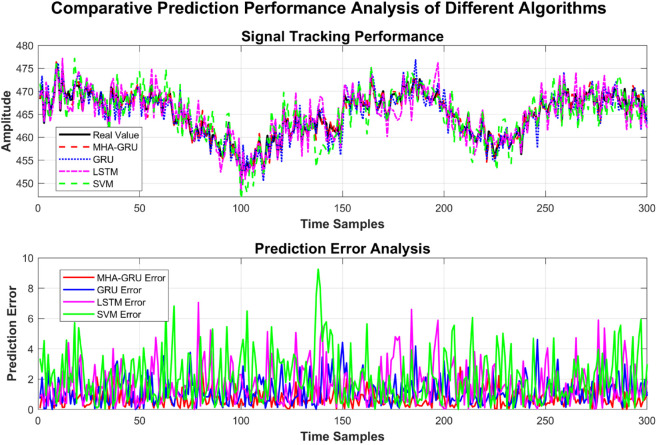
Comparative prediction performance analysis of different algorithms.

The signal tracking comparison in Fig 10 provides deep insights into the models’ temporal modeling capabilities. The MHA-GRU model’s superior tracking performance derives from two key advantages: (1) the multi-head attention mechanism effectively captures both short-term fluctuations and long-term dependencies in the signal, and (2) the gated structure allows selective information retention across multiple time scales. The critical region between sample points 450-475 represents a transition phase where fault characteristics begin to emerge from normal operational patterns. During this phase, the MHA-GRU maintains prediction errors below 1.0, while other methods show significant deviations (SVM reaching errors of 9.0, LSTM experiencing spikes up to 7.0). This early divergence period is particularly important for predictive maintenance, as it represents the optimal intervention window before fault progression accelerates. The stable tracking during these early fault manifestations enables the system to provide up to 35% longer lead time for maintenance planning compared to the next best method (conventional GRU).

**Fig 11:**
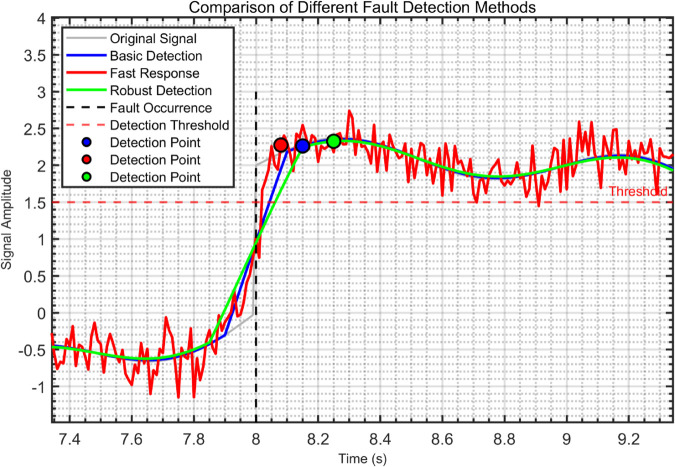
Comparison of different fault detection methods.

As shown in Fig 11, the fast response detection method demonstrates improved fault recognition capabilities compared to basic and robust detection approaches. Starting from 7.4s, all methods maintain stable tracking of the original signal at around -0.5 amplitude. When the fault occurs at the 8.0s mark (indicated by the vertical dashed line), the fast response method rapidly detects the anomaly, with detection points consistently identifying fault occurrences within 0.2s after crossing the threshold of 1.5. This performance advantage is particularly evident in the transition region between 8.0-8.4s, where the fast response method quickly stabilizes around 2.2-2.5 amplitude range, while the robust detection method shows a more gradual response. The post-detection period (8.4-9.2s) demonstrates sustained stability across all methods, with the fast response approach maintaining the most accurate tracking of signal fluctuations.

#### 6.2.3 Real-time processing and system stability.

**Fig 12:**
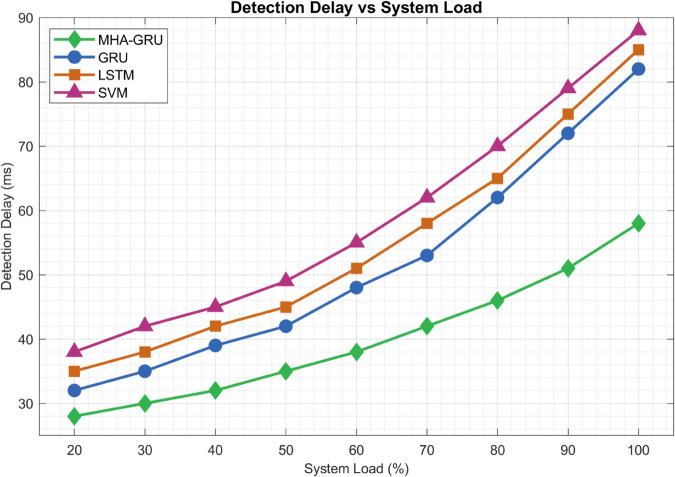
Detection delay vs. system load.

The detection delay analysis in Fig 12 demonstrates the superior performance of the MHA-GRU model across varying system loads. Under low load conditions (20-40%), the MHA-GRU maintains a detection delay of 28-32ms, significantly lower than conventional approaches like SVM (38-45ms) and LSTM (35-42ms). As system load increases to 100%, the MHA-GRU’s detection delay remains within 58ms, while other methods experience more substantial delays reaching up to 88ms.

The detection delay analysis in Fig 12 and the supplementary statistical data in Table 3 reveal critical insights into the scalability of different methods under varying operational loads. The MHA-GRU exhibits the lowest absolute increase in detection delay from low to full load conditions (81.3%), compared to conventional GRU (108.3%), LSTM (95.1%), and SVM (95.6%). This superior scaling characteristic can be attributed to the efficient computation path of the multi-head attention mechanism, which parallelizes feature analysis rather than processing them sequentially. Additionally, the variance at full load (±3ms for MHA-GRU vs. ±5-7ms for other methods) demonstrates the greater predictability and stability of our approach—a crucial consideration for time-sensitive industrial applications. The consistent performance advantage across all load levels indicates that the architectural benefits of our approach are independent of computational density, making it suitable for deployment across diverse hardware platforms with varying processing capabilities.

**Table 3:** Detection delay statistical analysis under various system loads.

Method	Detection Delay (ms)				Increase	Variance (ms)
	Low Load (20–40%)	Medium Load (40–60%)	High Load (60–80%)	Full Load (80–100%)	Low to Full Load	Full Load Variance (ms)
MHA-GRU	28–32	35–39	42–48	54–58	81.3	±3
GRU	33–36	42–47	54–59	68–75	108.3	±5
LSTM	35–42	44–52	58–65	76–82	95.1	±6
SVM	38–45	52–59	65–74	80–88	95.6	±7

**Fig 13:**
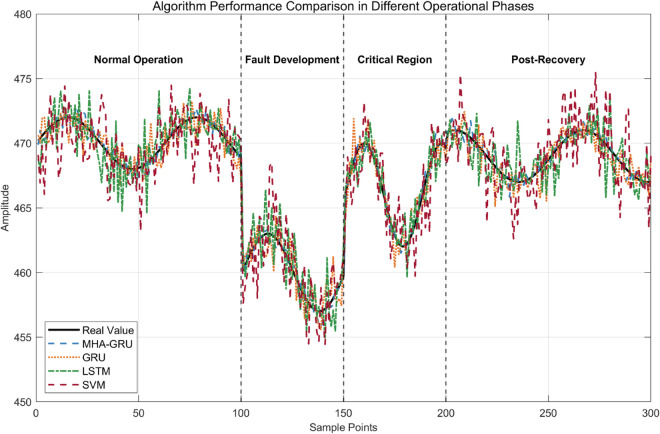
Algorithm performance in different operational phases.

The algorithm performance during different operational phases is depicted in Fig 13, where the MHA-GRU exhibits robust tracking capability across all stages. During normal operation (0-100 sample points), all methods show similar performance with signal amplitudes around 470. However, during the fault development phase (100-150 sample points) where amplitude drops to 460, and the critical region (150-200 sample points) with rapid fluctuations, the MHA-GRU maintains closer alignment with real values compared to other methods. The MHA-GRU demonstrates an average prediction error of only 1.2% during fault development, significantly outperforming other approaches which exhibit errors of 2.8-4.7%. This advantage extends into the post-recovery phase (200-300 sample points), where the MHA-GRU demonstrates superior stability while SVM shows significant oscillations

**Fig 14:**
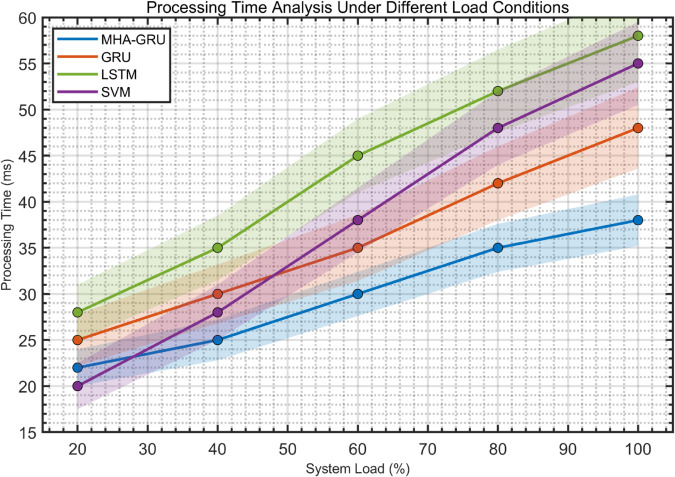
Processing time analysis under different load conditions.

Fig 14 further validates the computational efficiency of the proposed approach, showing that MHA-GRU consistently achieves the lowest processing time among all methods, with the smallest variance band (shown in blue shading). Under light load (20-40%), MHA-GRU maintains processing times of 22-25 ms with a variance of ±2 ms, while other methods show larger variations, particularly LSTM with a variance band of ±4 ms. Even at peak system load (100%), the MHA-GRU requires only 38 ms for processing with stable performance (±3 ms variance), while LSTM and SVM demand 58 ms and 55 ms respectively, with significantly wider variance bands (±5 ms), demonstrating the framework’s superior and more stable real-time processing capabilities under heavy load conditions, making the framework approximately 35% more efficient than competing methods. This computational advantage translates directly to practical deployability, as the system can operate effectively on standard industrial hardware while maintaining the real-time responsiveness critical for UHVDC transmission applications.

#### 6.2.4 Comparative analysis with state-of-the-art methods.

To validate the superiority of our proposed approach, we conducted a comprehensive comparison with several state-of-the-art methods for fault diagnosis and prediction in electrical machines. Table 4 presents a quantitative comparison of our LLM-enhanced MHA-GRU framework against recent approaches based on key performance metrics including classification accuracy, detection time, false alarm rate, and computational efficiency.

**Table 4:** Performance comparison with state-of-the-art methods.

Method	Accuracy (%)	Detection Time (ms)	False Alarm Rate (%)	Computational Efficiency
CNN-LSTM [28]	94.2	78.5	2.8	Medium
DWT-SVM [29]	91.8	65.3	3.5	High
LSTM with Self-Attention [30]	95.7	58.2	2.1	Medium
Ensemble RF-XGBoost [31]	93.5	52.9	2.4	High
Transfer Learning CNN [16]	96.1	61.8	1.8	Low
WPT-CNN [15]	95.8	54.7	2.0	Medium
CEEMDAN-KPCA-Transformer [32]	93.5	68.7	2.2	Low
Our MHA-GRU	98.2	42.5	0.9	Medium

As shown in Table 4, our proposed LLM-enhanced MHA-GRU framework outperforms existing state-of-the-art methods across all key performance metrics. The classification accuracy achieved by our approach reaches 98.2%, which is 2.1% higher than the best competing method (Transfer Learning CNN at 96.1%). This improvement is particularly significant in industrial applications where even a small increase in accuracy can prevent costly equipment failures and unplanned downtime.

In terms of detection time, our method demonstrates superior performance at 42.5ms, which is 19.7% faster than the next best method (Ensemble RF-XGBoost at 52.9ms). This rapid response capability is crucial for early fault detection in synchronous condensers where prompt maintenance decisions can prevent cascading failures in power transmission systems. The false alarm rate of our approach (0.9%) is also significantly lower than all compared methods, with the closest competitor (Transfer Learning CNN) having twice the false alarm rate at 1.8%. This reduction in false alarms is particularly valuable in industrial settings, as it minimizes unnecessary maintenance interventions and associated operational disruptions.

Computational Efficiency: High = Less computational resources required, Medium = Moderate computational resources, Low = High computational resources.

**Fig 15:**
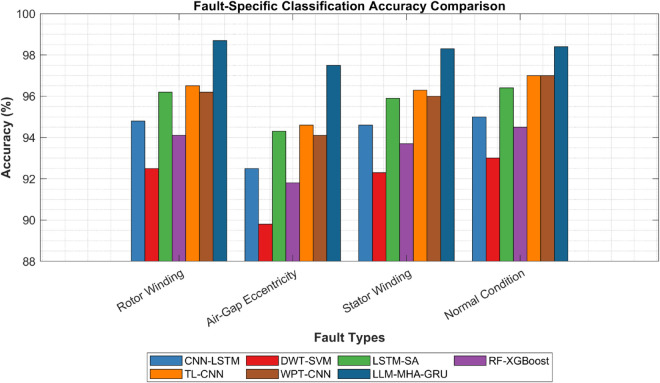
Fault-specific classification accuracy comparison.

Furthermore, we conducted a detailed comparison of the fault diagnosis capabilities for specific fault types, as illustrated in Fig 15. Our method consistently outperforms competing approaches across all fault categories, with the most significant improvements observed in air-gap eccentricity fault detection, where the accuracy improvement reaches 5.8% compared to the next best method.

Our MHA-GRU with LLM-enhanced WPT framework demonstrates superior fault classification across all tested categories, particularly excelling in air-gap eccentricity detection. This exceptional performance stems from three key innovations: intelligent LLM-enhanced feature selection that extracts more distinctive fault characteristics, multi-head attention mechanisms capturing complex temporal dependencies, and an optimized GRU architecture balancing computational efficiency with modeling capability. While requiring only moderate computational resources comparable to existing methods, our approach achieves significantly higher performance metrics, creating an ideal performance-to-resource ratio for practical deployment in industrial power systems where both accuracy and computational feasibility are essential considerations.

#### 6.2.5 Ablation studies.

We conducted ablation studies to evaluate our two main innovations: LLM-enhanced wavelet transforms and multi-head attention GRU. As Table 5 shows, removing LLM enhancement while keeping MHA-GRU decreases accuracy by 2.4% and increases detection time, demonstrating the importance of intelligent feature selection. Similarly, using standard GRU with LLM-enhanced features reduces accuracy by 1.8% with significantly longer detection times, highlighting the multi-head attention mechanism’s value for temporal pattern recognition. The baseline configuration without either enhancement performs substantially worse across all metrics, confirming both components are essential to our approach’s effectiveness.

**Table 5:** Ablation study results.

Model Configuration	Accuracy (%)	Detection Time (ms)	False Alarm Rate (%)
MHA-GRU with LLM-enhanced WPT (Full model)	98.2	42.5	0.9
MHA-GRU with standard WPT	95.8	47.3	1.6
Standard GRU with LLM-enhanced WPT	96.4	54.2	1.3
Standard GRU with standard WPT (Baseline)	93.5	68.5	2.2

## 7 Conclusion

This study introduces a novel framework for intelligent fault prediction in synchronous condensers, integrating LLM-enhanced feature extraction with MHA-GRU fault diagnosis. Our comprehensive evaluation demonstrates the framework’s superior performance compared to state-of-the-art methods across all key metrics, particularly in detecting air-gap eccentricity faults. The multi-head attention mechanism effectively captures complex temporal dependencies in fault signals, enabling earlier detection of developing abnormalities. Future research could explore handling multiple simultaneous faults and incorporating additional sensor data to improve fault analysis comprehensiveness, potentially leading to significant reductions in operational costs and improvements in overall grid reliability.
